# A patient-driven registry on Behçet’s disease: the AIDA for patients pilot project

**DOI:** 10.3389/fmed.2023.1188021

**Published:** 2023-06-16

**Authors:** Carla Gaggiano, Alessandra Del Bianco, Jurgen Sota, Stefano Gentileschi, Piero Ruscitti, Roberto Giacomelli, Matteo Piga, Francesca Crisafulli, Sara Monti, Giacomo Emmi, Amato De Paulis, Antonio Vitale, Maria Tarsia, Valeria Caggiano, Rossana Nuzzolese, Veronica Parretti, Claudia Fabiani, Giuseppe Lopalco, Armin Maier, Marco Cattalini, Donato Rigante, Marcello Govoni, Francesca Li Gobbi, Serena Guiducci, Paola Parronchi, Achille Marino, Francesco Ciccia, Maria Cristina Maggio, Emma Aragona, Elena Bartoloni, Annamaria Iagnocco, Ombretta Viapiana, Gian Domenico Sebastiani, Silvana Guerriero, Antonella Insalaco, Emanuela Del Giudice, Giovanni Conti, Patrizia Barone, Alma Nunzia Olivieri, Antonio Brucato, Francesco Carubbi, Paola Triggianese, Angela Mauro, Gian Marco Tosi, Alex Fonollosa, Henrique Ayres Mayrink Giardini, Gaafar Ragab, Samar Tharwat, José Hernández-Rodríguez, Petros P. Sfikakis, Katerina Laskari, Anastasios Karamanakos, Gerard Espinosa, Farhad Shahram, Haner Direskeneli, Andrea Hinojosa-Azaola, Daniela Opris-Belinski, Ibrahim A. AlMaghlouth, Gülen Hatemi, Mehmet Akif Eksin, Fatos Önen, Ewa Więsik-Szewczyk, Nurullah Akkoç, Abdurrahman Tufan, Ali Şahin, Şükran Erten, Seza Ozen, Ezgi Deniz Batu, Bruno Frediani, Alberto Balistreri, Luca Cantarini

**Affiliations:** ^1^Rheumatology Unit, Department of Medical Sciences, Surgery and Neurosciences, University of Siena and Azienda Ospedaliero-Universitaria Senese [European Reference Network (ERN) for Rare Immunodeficiency, Autoinflammatory, and Autoimmune Diseases (RITA) Center], Siena, Italy; ^2^S.I.M.B.A (Associazione Italiana Sindrome e Malattia di Behçet), Pontedera, Italy; ^3^Rheumatology Unit, Department of Biotechnological & Applied Clinical Sciences, University of L’Aquila, L’Aquila, Italy; ^4^Rheumatology and Immunology Unit, Department of Medicine, University of Rome “Campus Biomedico”, Rome, Italy; ^5^Rheumatology Unit, Department of Medical Sciences, University and AOU of Cagliari, Cagliari, Italy; ^6^Rheumatology and Clinical Immunology, Department of Clinical and Experimental Sciences, University of Brescia and Spedali Civili [European Reference Network (ERN) for Rare Immunodeficiency, Autoinflammatory, and Autoimmune Diseases (RITA) Center], Brescia, Italy; ^7^Division of Rheumatology, Fondazione IRCCS Policlinico San Matteo [European Reference Network (ERN) for Rare Immunodeficiency, Autoinflammatory and Autoimmune Diseases (RITA) Center], Pavia, Italy; ^8^Department of Experimental and Clinical Medicine, University of Florence, Florence, Italy; ^9^Centre for Inflammatory Diseases, Department of Medicine, Monash Medical Centre, Monash University, Clayton, VIC, Australia; ^10^Department of Translational Medical Sciences, Section of Clinical Immunology, University of Naples Federico II, Naples, Italy; ^11^Center for Basic and Clinical Immunology Research (CISI), WAO Center of Excellence, University of Naples Federico II, Naples, Italy; ^12^Clinical Paediatrics, Department of Molecular Medicine and Development, University of Siena and Azienda Ospedaliero-Universitaria Senese [European Reference Network (ERN) for Rare Immunodeficiency, Autoinflammatory, and Autoimmune Diseases (RITA) Center], Siena, Italy; ^13^Ophthalmology Unit, Department of Medicine, Surgery and Neurosciences, University of Siena and Azienda Ospedaliero-Universitaria Senese [European Reference Network (ERN) for Rare Immunodeficiency, Autoinflammatory, and Autoimmune Diseases (RITA) Center], Siena, Italy; ^14^Rheumatology Unit, Department of Emergency and Organ Transplantation, University of Bari, Bari, Italy; ^15^Rheumatology Unit, Department of Medicine, Central Hospital of Bolzano, Bolzano, Italy; ^16^Pediatric Clinic, University of Brescia and Spedali Civili di Brescia [European Reference Network (ERN) for Rare Immunodeficiency, Autoinflammatory, and Autoimmune Diseases (RITA) Center], Brescia, Italy; ^17^Department of Life Sciences and Global Health, Fondazione Policlinico Universitario A. Gemelli IRCCS, Rome, Italy; ^18^Rare Diseases and Periodic Fevers Research Centre, Università Cattolica del Sacro Cuore, Rome, Italy; ^19^Rheumatology Unit, Department of Medical Sciences, Azienda Ospedaliero-Universitaria S. Anna-Ferrara, University of Ferrara, Ferrara, Italy; ^20^Rheumatology Unit, Hospital S. Giovanni di Dio, Azienda USL-Toscana Centro, Florence, Italy; ^21^Immunology and Cellular Therapies Unit, University Hospital Careggi, Florence, Italy; ^22^Unit of Pediatric Rheumatology, Azienda Socio-Sanitaria Territoriale (ASST) Gaetano Pini-Centro Specialistico Ortopedico Traumatologico (CTO), Milan, Italy; ^23^Department of Precision Medicine, Università Degli Studi Della Campania Luigi Vanvitelli, Naples, Italy; ^24^University Department of Health Promotion, Mother and Child Care, Internal Medicine and Medical Specialties (PROMISE) “G. D’Alessandro”, University of Palermo, Palermo, Italy; ^25^Division of Gastroenterology, Ospedali Riuniti Villa Sofia-Vincenzo Cervello, Palermo, Italy; ^26^Rheumatology Unit, Department of Medicine, University of Perugia, Perugia, Italy; ^27^Academic Rheumatology Center, Dipartimento Scienze Cliniche e Biologiche, Università degli Studi di Torino, Turin, Italy; ^28^Rheumatology Unit, Department of Medicine, University of Verona, Verona, Italy; ^29^Rheumatology Unit, San Camillo – Forlanini Hospital, Rome, Italy; ^30^Department of Ophthalmology and Otolaryngology, University of Bari, Bari, Italy; ^31^Division of Rheumatology, Ospedale Pediatrico Bambino Gesù, IRCCS [European Reference Network (ERN) for Rare Immunodeficiency, Autoinflammatory and Autoimmune Diseases (RITA) Center], Rome, Italy; ^32^Pediatric and Neonatology Unit, Department of Maternal Infantile and Urological Sciences, Sapienza University of Rome, Latina, Italy; ^33^Pediatric Nephrology and Rheumatology Unit, Azienda Ospedaliero Universitaria (AOU) G Martino, Messina, Italy; ^34^Department of Clinical and Experimental Medicine, University of Catania, Catania, Italy; ^35^Department of Woman, Child and of General and Specialized Surgery, University of Campania “Luigi Vanvitelli”, Naples, Italy; ^36^Department of Biomedical and Clinical Sciences, Fatebenefratelli Hospital, Università di Milano, Milan, Italy; ^37^Department of Life, Health & Environmental Sciences and Internal Medicine and Nephrology Unit, Department of Medicine, University of L’Aquila and ASL Avezzano-Sulmona-L’Aquila, San Salvatore Hospital, L’Aquila, Italy; ^38^Rheumatology, Allergology and Clinical Immunology, Department of System Medicine, University of Rome Tor Vergata, Rome, Italy; ^39^Pediatric Rheumatology Unit, Department of Childhood and Developmental Medicine, Fatebenefratelli-Sacco Hospital, Milan, Italy; ^40^Department of Ophthalmology, Biocruces Bizkaia Health Research Institute, Cruces University Hospital, University of the Basque Country, Barakaldo, Spain; ^41^Rheumatology Division, Hospital das Clinicas (HCFMUSP), Faculdade de Medicina, Universidade de São Paulo, São Paulo, Brazil; ^42^Rheumatology and Clinical Immunology Unit, Department of Internal Medicine, Faculty of Medicine, Cairo University, Giza, Egypt; ^43^Faculty of Medicine, New Giza University, Giza, Egypt; ^44^Rheumatology and Immunology Unit, Internal Medicine Department, Mansoura University, Mansoura, Egypt; ^45^Department of Internal Medicine, Faculty of Medicine, Horus University, New Damietta, Egypt; ^46^Department of Autoimmune Diseases, Institut d’Investigacions Biomèdiques August Pi I Sunyer (IDIBAPS), Hospital Clínic of Barcelona [European Reference Network (ERN) for Rare Immunodeficiency, Autoinflammatory and Autoimmune Diseases (RITA) Center], University of Barcelona, Barcelona, Spain; ^47^Joint Academic Rheumatology Program, Medical School, National and Kapodistrian University of Athens, Athens, Greece; ^48^Department of Rheumatology, Evangelismos General Hospital, Athens, Greece; ^49^Behcet’s Disease Unit, Rheumatology Research Center, Shariati Hospital Tehran University of Medical Sciences, Tehran, Iran; ^50^Department of Internal Medicine, Division of Rheumatology, Marmara University, Faculty of Medicine, Istanbul, Turkey; ^51^Department of Immunology and Rheumatology, Instituto Nacional de Ciencias Médicas Y Nutrición Salvador Zubirán, Mexico City, Mexico; ^52^Rheumatology and Internal Medicine Department, Carol Davila University of Medicine and Pharmacy, Bucharest, Romania; ^53^Rheumatology Unit, Department of Medicine, College of Medicine, King Saud University, Riyadh, Saudi Arabia; ^54^College of Medicine Research Center, College of Medicine, King Saud University, Riyadh, Saudi Arabia; ^55^Department of Internal Medicine, Division of Rheumatology, Cerrahpasa Medical School, Istanbul University-Cerrahpasa, Istanbul, Turkey; ^56^Behçet’s Disease Research Center, Istanbul University-Cerrahpasa, Istanbul, Turkey; ^57^Rheumatology Clinic, Ankara City Hospital, Ankara, Turkey; ^58^Department of Internal Medicine Division of Rheumatology, Dokuz Eylül University Faculty of Medicine, İzmir, Turkey; ^59^Department of Internal Medicine, Pneumonology, Allergology and Clinical Immunology, Central Clinical Hospital of the Ministry of National Defense, Military Institute of Medicine, National Research Institute, Warsaw, Poland; ^60^Division of Rheumatology, Department of Internal Medicine, School of Medicine, Manisa Celal Bayar University, Manisa, Turkey; ^61^Division of Rheumatology, Department of Internal Medicine, Faculty of Medicine Gazi University, Ankara, Turkey; ^62^Division of Rheumatology, Department of Internal Medicine, Sivas Cumhuriyet University Medical Faculty, Sivas, Turkey; ^63^Pediatric Rheumatology Unit, Department of Pediatrics, Hacettepe University School of Medicine, Ankara, Turkey; ^64^Bioengineering and Biomedical Data Science Lab, Department of Medical Biotechnologies, University of Siena, Siena, Italy

**Keywords:** Behçet’s disease, patient-driven registries, rare diseases, autoinflammatory diseases, patient involvement, patient-reported outcomes

## Abstract

**Introduction:**

This paper describes the creation and preliminary results of a patient-driven registry for the collection of patient-reported outcomes (PROs) and patient-reported experiences (PREs) in Behçet’s disease (BD).

**Methods:**

The project was coordinated by the University of Siena and the Italian patient advocacy organization SIMBA (Associazione Italiana Sindrome e Malattia di Behçet), in the context of the AIDA (AutoInflammatory Diseases Alliance) Network programme. Quality of life, fatigue, socioeconomic impact of the disease and therapeutic adherence were selected as core domains to include in the registry.

**Results:**

Respondents were reached via SIMBA communication channels in 167 cases (83.5%) and the AIDA Network affiliated clinical centers in 33 cases (16.5%). The median value of the Behçet’s Disease Quality of Life (BDQoL) score was 14 (IQR 11, range 0–30), indicating a medium quality of life, and the median Global Fatigue Index (GFI) was 38.7 (IQR 10.9, range 1–50), expressing a significant level of fatigue. The mean Beliefs about Medicines Questionnaire (BMQ) necessity-concern differential was 0.9 ± 1.1 (range – 1.8–4), showing that the registry participants prioritized necessity belief over concerns to a limited extent. As for the socioeconomic impact of BD, in 104 out of 187 cases (55.6%), patients had to pay from their own pocket for medical exams required to reach the diagnosis. The low family socioeconomic status (*p* < 0.001), the presence of any major organ involvement (*p* < 0.031), the presence of gastro-intestinal (*p* < 0.001), neurological (*p* = 0.012) and musculoskeletal (*p* = 0.022) symptoms, recurrent fever (*p* = 0.002), and headache (*p* < 0.001) were associated to a higher number of accesses to the healthcare system. Multiple linear regression showed that the BDQoL score could significantly predict the global socioeconomic impact of BD (*F* = 14.519, OR 1.162 [CI 0.557–1.766], *p* < 0.001).

**Discussion:**

Preliminary results from the AIDA for Patients BD registry were consistent with data available in the literature, confirming that PROs and PREs could be easily provided by the patient remotely to integrate physician-driven registries with complementary and reliable information.

## 1. Introduction

Patient-driven or patient self-reported registries are organized systems collecting uniform data directly from patients to evaluate specified outcomes in a defined population (1). They integrate the classical physician-driven data collection with patient-reported outcomes (PROs) and patient-reported experiences (PREs), adding invaluable contents to research studies. They are also expected to improve the doctor-patient relationship, building trust and mutual connection through the patient’s transition from passive to active participant in all the steps of clinical research. When based on user-friendly electronical records accessible online via remote devices, they allow the widest participation even among people with disabilities or living far from the research center, ensuring that geographical and social inequalities are overcome.

The AIDA Network has been established in 2020 as a collaborative framework for international research on autoinflammatory diseases and ocular immune-mediated diseases, with more than 170 clinical sites worldwide.^1^ As one of its main efforts, the AIDA Network Registries action led to the development of nine clinical registries, all of them being physician-driven (ClinicalTrials.gov Identifier: NCT05200715). In this context, since the beginning of the project, international experts from the AIDA Network made strategic collaborations with national patient advocacy organizations (PAOs) sharing the programme goals and vision. Among these, there is the development of a patient-driven registry named “AIDA for Patients” covering the whole spectrum of diseases under surveillance and declined in all the national languages spoken in the Network, to complement data collection with PROs and PREs directly entered by patients.

This paper is aimed at describing methods and preliminary results of the AIDA for Patients pilot project, a patient-driven registry for Italian persons affected by Behçet’s disease (BD) and their caregivers, which has been developed in collaboration with the Italian PAO SIMBA (Associazione Italiana Sindrome e Malattia di Behçet, https://www.behcet.it/). The registry data were preliminarily analyzed to evaluate the quality of life, fatigue level and therapeutic adherence of people affected by BD, and the socioeconomic impact of the disease in Italy.

## 2. Methods

### 2.1. Registry development

The AIDA for Patients registry is hosted by the REDCap platform (Research Electronic Data Capture, https://projectredcap.org), a secure web application designed to support data capture for research studies. Data were entered into electronic forms directly by the participants, recruited through SIMBA communication channels (mailing list and social media) and the AIDA Network affiliated clinical centers in Italy. Participants were able to access the registry through their mobile devices or computers via a QR code or a web link to the REDCap homepage of the project. They were initially screened for inclusion through a short survey addressing the respondents to 7 different profiles: (1) adult patient >17 year-old, (2) pediatric patient 13- to 17 year-old, (3) pediatric patient 8- to 12 year-old, (4) 13- to 17 year-old patient’s parent, (5) 8- to 12 year-old patient’s parent, (6) 5- to 7 year-old patient’s parent, and (7) 2- to 4 year-old patient’s parent. Respondents were automatically excluded by the system if the diagnosis of BD was only suspected or under evaluation, and in case of parents of <2 year-old patients. Each profile comprised 3 to 5 data collection instruments appropriate to the age and role of the participant, which overall required about 10 min for completion.

The core domains addressed by the registry were identified through a literature analysis, including also the Omeract Core Set of Domains for Outcome Measures in Behçet’s Syndrome (2), and discussed among a panel of BD experts and patients’ representatives from SIMBA. They included quality of life, fatigue, socioeconomic impact of the disease and therapeutic adherence. Three domains were investigated through validated questionnaires in the Italian language: Behçet’s Disease Quality of Life (BDQoL) (3), Beliefs about Medicines Questionnaire (BMQ) (4), PedsQLcore (5), PedsQLfatigue (5), and Multidimensional Assessment of Fatigue (MAF) (6). The BDQoL score has a 0–30 validity range, where higher scores indicate lower quality of life in adults. The PedsQLcore score has a 0–100 validity range, with higher scores meaning better quality of life in children aged 2–18 years. The BMQ questionnaire is made up of two sections: the BMQ concern (BMQc), which investigates the strength of concerns about the safety of specific medications taken by the subject for BD, and the BMQ necessity (BMQn), which measures how much the subject feels important to take the specific medications prescribed for BD. Both sections have a 1–5 validity range, with higher scores indicating stronger beliefs; the BMQ necessity-concern differential has a – 4–+4 validity range, indicating that necessity exceeds concern if the differential is >0, or concern exceeds necessity if <0. The global fatigue index (GFI) resulting from the MAF questionnaire ranges 0–50, where a higher index indicates more severe fatigue in adults. The PedQLfatigue score has a 0–100 validity range, with a higher score meaning less severe fatigue in children aged 2–18 years. On the other hand, a new questionnaire (including 10 to 20 items according to the age and role of the respondent) was specifically developed by the authors and approved by SIMBA representatives to investigate the patient’s diagnostic journey and socioeconomic impact of the disease. The family socioeconomic status was defined “average” when the subject stated “I earn enough money to meet the needs of my family,” “poorer than average” if the answer was “my financial situation is troublesome” and “healthier than average” in case of the answer “I lead a very comfortable life.” The total number of accesses to medical services resulted from the sum of the number of accesses to the general practitioner, the emergency department and the specialistic services in the last 3 months. The social burden index (SBI) resulted from the sum of the days lost at work/school by the subject and by his/her relatives due to BD and the number of days of hospitalization in the previous 3 months. The total socioeconomic impact for each subject was calculated as the sum of the total number of accesses to medical services and the SBI.

The study protocol conformed to the tenets of the Declaration of Helsinki and was approved by the local Ethics Committee of the University of Siena (Reference No. 14951). Informed consent for using clinical data for research purposes was obtained electronically at the start of the pre-screening survey via the following statement in the Italian language “By clicking this button, you are expressing your willing to participate in this survey study and voluntarily give your consent.” Patients were informed by the physician or through the accompanying message of invitation that their personal information would be separated from their clinical data by using a pseudonym. The researcher who handled clinical data and performed statistical analysis had no access to the mailing list of the subjects invited by SIMBA nor to any personal information potentially capable to identify the subjects. On the other hand, the representatives from SIMBA and the treating physicians who invited the possible candidates had no access to the clinical data entered by the participants.

### 2.2. Statistical analysis

Statistical analysis was performed by using JASP open-source statistics package version 0.16.3. Descriptive statistics included sample sizes, mean and standard deviation or median and interquartile range (IQR), as appropriate. Shapiro–Wilk test was used to assess normality distribution of data. Differences in continuous data between independent groups were compared by Mann–Whitney U test or Kruskal–Wallis H test with Dunn’s post-hoc test. Relationships between continuous variables failing to meet parametric assumptions were tested through Spearman’s rho (*ρ*). Multiple regression analysis was used to predict outcomes of multiple continuous variables (95% CI). The threshold for statistical significance was set to *p* < 0.05 and all *p*-values were two-sided.

## 3. Results

During the period from March to October 2022, 200 participants (M:F = 1:2.5) entered the registry. Respondents were reached via SIMBA communication channels in 167 cases (83.5%) and the AIDA Network affiliated clinical centers in 33 cases (16.5%). Out of 200 respondents, 187 fulfilled inclusion criteria and were able to enter data into the registry as patients (*n* = 180) or patients’ parents (*n* = 7); the remaining 13 respondents were excluded by the system because the diagnosis of BD in the participant (*n* = 4) or in the participant’s child (*n* = 3) was not confirmed by a physician, or for other reasons (*n* = 6). The median age of affected subjects was 43 years (IQR 17, range 18–69) for adults and 15 years (IQR 3.5, range 9–16) for children. The median disease duration was 13 years (IQR 15, range 1–54), the median diagnostic delay was 4 years (IQR 7.8, range 0–48). There was a negative correlation with large effect size between the diagnostic delay and the year of disease onset (*ρ* = −0.72, *p* < 0.001).

Descriptive clinical and socioeconomic information of the participants is provided in Table 1.

**Table 1:** Descriptive clinical and socioeconomic information of the participants.

BD-related manifestations experienced by the participants anytime during the clinical history			
Oral ulcers	137 (73.3%)	Recurrent fever	73 (39.0%)
Articular manifestations	113 (60.4%)	Ocular involvement	60 (32.1%)
Headache	99 (52.9%)	Neurological manifestations	52 (27.8%)
Genital ulcers	93 (49.7%)	Vascular thrombosis	37 (19.8%)
Cutaneous manifestations	89 (47.6%)	Axial arthritis	35 (18.7%)
Gastro-intestinal manifestations	79 (42.2%)		
Major organ involvement (ocular, neurological excluding headache, gastro-intestinal, vascular)			
Yes 120 (64.2%)			
No 26 (13.9%)			
Missing 41 (21.9%)			
BMI classification		Regular physical exercise	
Normal weight	71 (37.9%)	Yes	29 (15.5%)
Overweight	39 (20.9%)	No	114 (60.9%)
Obese	21 (11.2%)	Missing	44 (23.5%)
Underweight	11 (5.9%)		
Missing	45 (24.1%)		
N. of years in school		Socioeconomic status	
0–8	10 (5.3%)	Healthier than average	10 (5.3%)
9–13	49 (26.2%)	Average	62 (33.2%)
14–18	54 (28.9%)	Poorer than average	44 (23.5%)
>18	30 (16.0%)	Missing	71 (38.0%)
Missing	44 (23.5%)		
Median N. of specialistic centers visited before the diagnosis			
3 (IQR 3, range 0–21)			
Necessity to pay for medical exams^*^		Disease information at diagnosis	
Yes	104 (55.6%)	By medical professionals	109 (58.3%)
No	37 (19.8%)	By patient associations	8 (4.3%)
Missing	45 (24.1%)	Autonomous	25 (13.4%)

BMI, body mass index; BD, Behçet’s disease; IQR, interquartile range. ^*^Medical consults, laboratory, and radiological exams, genetic tests or other procedures required during the diagnostic journey up to the diagnosis of Behçet’s disease.

### 3.1. Quality of life

**Figure 1:**
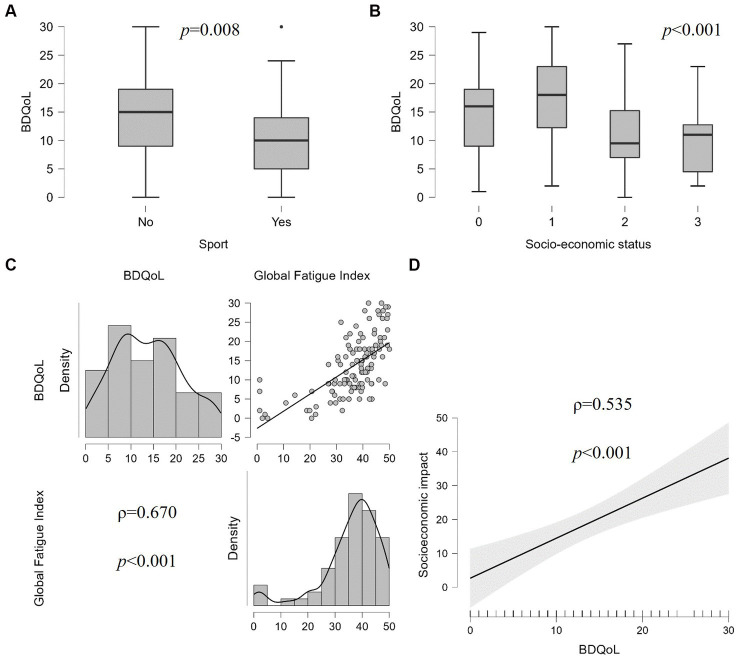
Correlation of the quality-of-life variation – measured by Behçet’s Disease Quality of Life (BDQoL) questionnaire – and **(A)** sport habit, **(B)** socioeconomic status of the family [0 = not disclosed; 1 = poorer than average; 2 = average; 3 = healthier than average], **(C)** fatigue level measured by Multidimensional Assessment of Fatigue (MAF), and **(D)** global socioeconomic impact of the disease.

The median value of BDQoL at the time of the survey completion was 14 (IQR 11, range 0–30) and the mean score of PedQLCore was 61.5 ± 24.3 (range 40.3–85.8). The median value of BDQoL was higher in patients showing cutaneous (*p* = 0.029), gastro-intestinal (*p* < 0.001), neurological (*p* = 0.002), musculoskeletal (*p* < 0.001) symptoms and headache (*p* = 0.004). The median value of BDQoL was higher in patients not practicing any sport (median 15, IQR 10 versus 10, IQR 9, *p* = 0.008) and positively correlated with the BMI value with small effect (*ρ* = 0.28, *p* < 0.001) as shown in Figure 1A. Subjects defining their socioeconomic status as poorer than average had higher values of BDQoL (median 18, IQR 10.8) compared to average (median 9.5, IQR 8.3, *p* < 0.001) and healthier than average (median 11, IQR 8.3, *p* = 0.002), as shown in Figure 1B. Also, a positive correlation was found between BDQoL value and the number of specialistic centers visited before the diagnosis (*ρ* = 0.24, *p* = 0.005), the number of accesses to medical services in the previous 3 months (*ρ* = 0.58, *p* < 0.001), the social burden index (*ρ* = 0.40, *p* < 0.001), the GFI (*ρ* = 0.67, *p* < 0.001) (Figure 1C), the BMQn score (*ρ* = 0.19, *p* < 0.031) and the BMQc score (*ρ* = 0.38, *p* < 0.001). Multiple linear regression using backward data entry showed that GFI (OR 0.43 [CI 0.17–0.68]), the number of accesses to medical services in the previous 3 months (OR 0.59 [CI 0.04–1.14]) and BMQn score (OR −3.01 [CI −6.36–0.33]) could significantly predict BDQoL (*F* = 12.95, *p* < 0.001).

### 3.2. Fatigue

The median GFI was 38.7 (IQR 10.9, range 1–50) and the mean PedQLFatigue total score was 63.9 ± 32.9 (range 31.9–100). The median value of GFI was higher in patients not practicing any sport (median 39, IQR 10.4 versus 36.5, IQR 11.8, *p* = 0.022) and in patients complaining of gastro-intestinal (*p* < 0.001), neurological (*p* = 0.048) and musculoskeletal (*p* = 0.015) symptoms, and headache (*p* < 0.001). Subjects estimating the socioeconomic status of their family as poorer than average had higher values of GFI (median 39.4, IQR 12.5) compared to average (median 37.1, IQR 10.9, *p* = 0.009) and healthier than average (median 35.9, IQR 29.6, *p* = 0.028). Also, participants who autonomously searched for information about their disease showed higher values of GFI than those receiving information from medical professionals (43.2, IQR 9.9 versus 37.5, IQR 9.9, *p* = 0.009). A positive correlation was also found between GFI value and the number of accesses to medical services in the previous 3 months (*ρ* = 0.43, *p* < 0.001), the SBI (*ρ* = 0.34, *p* < 0.001), the BMQn score (*ρ* = 0.24, *p* = 0.008), and the BMQc score (*ρ* = 0.29, *p* < 0.001).

### 3.3. Therapeutic adherence

The mean BMQn score was 4.1 ± 0.7 (range 1.4–5), the mean BMQc score 3.2 ± 0.8 (range 1–5) and the mean BMQ necessity-concern differential 0.9 ± 1.1 (range – 1.8–4). Subjects with major organ involvement had higher values of BMQn score than those with only minor BD manifestations (median 4.2, IQR 1 versus 3.8, IQR 0.6, *p* = 0.019). Participants with more than 18 school years had higher values of BMQn score (median 4.6, IQR 1) than those with 14–18 school years (median 4, IQR 1, *p* = 0.003) and 9–13 school years (median 4, IQR 0.8, *p* = 0.01). Subjects with a socioeconomic status defined as poorer than average had higher BMQc score (median 3.4, IQR 1.2) compared to average (median 3, IQR 1, *p* = 0.003) and healthier than average (median 2.7, IQR 0.5, *p* = 0.005). In addition, a positive correlation was found between both the BMQn and BMQc score and the number of accesses to medical services in the previous 3 months (*ρ* = 0.24, *p* = 0.006 and *ρ* = 0.20, *p* = 0.023, respectively) and between the BMQn score and the SBI (*ρ* = 0.29, *p* = 0.001).

### 3.4. Socioeconomic impact of the disease

**Figure 2:**
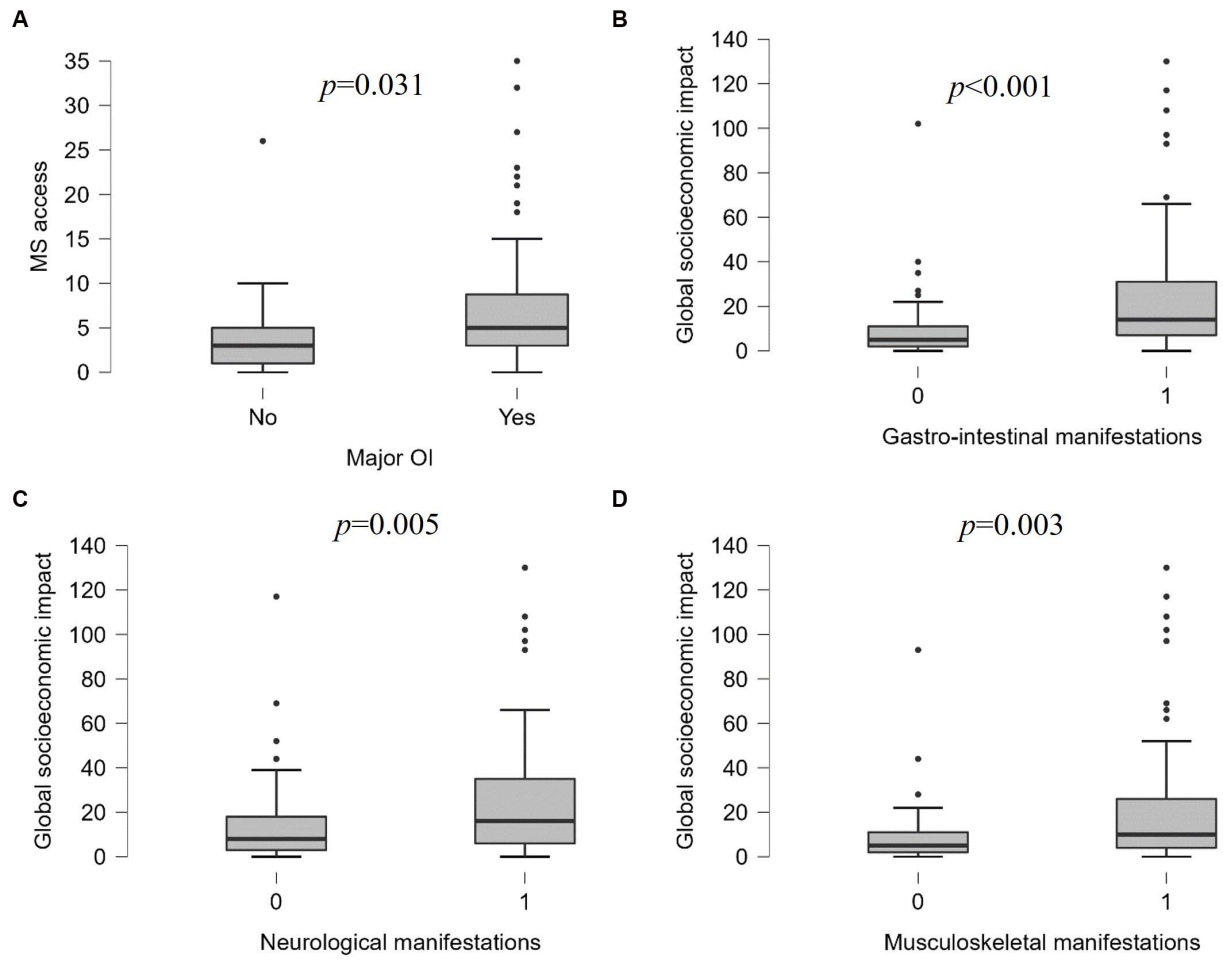
Socioeconomic impact of different clinical manifestations of Behçet’s disease: **(A)** number of medical service (MS) accesses in the last 3 months in patients with or without major organ involvement (OI); global socioeconomic impact of the disease in patients with or without gastro-intestinal **(B)**, neurological **(C)**, and musculoskeletal **(D)** manifestations.

During the previous 3 months, the median number of accesses to medical services was 4.5 (IQR 6.0, range 0–35): median 2 (IQR 3) visits to the general practitioner (range 0–51), median 0 (IQR 0) visits to the emergency department (range 0–51), median 2 (IQR 3) specialistic visits (range 0–20). The median SBI was 4.0 (IQR 15, range 0–120): median 2 (IQR 10) days lost at work (range 0–90), median 0 (IQR 3) days lost at work by relatives (range 0–30), median 0 (IQR 0) days of hospital admission (range 0–40). Overall, subjects with major organ involvement had a higher number of medical services accesses (median 5, IQR 5.75) and a higher SBI (median 4, IQR 24.3) than those with only minor BD manifestations (median 3, IQR 4, *p* = 0.031, and median 0, IQR 8, *p* = 0.012, respectively) (Figure 2A). A higher number of accesses to medical services was reported by participants with gastro-intestinal (*p* < 0.001), neurological (*p* = 0.012), musculoskeletal (*p* = 0.022) symptoms and those with recurrent fever (*p* = 0.002), and headache (*p* < 0.001), while subjects with gastro-intestinal symptoms (*p* < 0.001), recurrent fever (*p* = 0.015), and axial arthritis (*p* < 0.001) had higher SBI than those without these manifestations. The global socioeconomic impact of different clinical manifestations of BD is displayed in Figures 2B–D. Also subjects defining their socioeconomic status as poorer than average accessed medical services more frequently (median 6.5, IQR 6.7) compared to average (median 3.5, IQR 4, *p* < 0.001) and healthier than average (median 4, IQR 3, *p* = 0.006). Multiple linear regression using backward data entry showed that the BDQoL score can significantly predict the global socioeconomic impact of BD (*F* = 14.519, OR 1.162 [CI 0.557–1.766], *p* < 0.001), as shown in Figure 1D.

## 4. Discussion

This paper describes the methodology and preliminary data of a patient-driven registry for Italian-speaking people affected by BD, which can be easily accessed online by patients of different age groups and their caregivers. Over a period of 8 months, the registry access link and QR code were emailed and posted on Facebook by SIMBA and advertised directly by physicians through an informative leaflet given to patients during routine follow-up visits. Preliminary statistics of the registry enrolment clearly show that the most promising channel is the non-medical one, with 83.5% of spontaneous enrolments via SIMBA channels versus 16.5% via medical professionals. This can be explained by the fact that direct email/Facebook access to the screening survey is more immediate than access through the QR code or link printed on the leaflet. In addition, the context of the hospital visit may not be ideal to capture the attention of the patient, who naturally focuses on information about his/her health condition, the examinations that are prescribed, and therapeutic changes. The physician may also find it challenging to recruit patients in the short timeframe of the follow-up visit. According to these insights, the AIDA for Patients recruitment strategy should be remodulated in the future, on one hand, by boosting the role of national PAOs and running a wider internet and social-media campaign, on the other, by generating automatic email invitations linked to the AIDA physician-driven registry records to match physician- and patient-reported data.

The age distribution of participants corresponded to the epidemiology of the disease, with a peak in the 4th and 5th decades of life and a very limited representation of children (7). Recruiting children was even more challenging because of a limited access to email and Facebook in the 8–17 age group [https://www.statista.com/statistics/376128/facebook-global-user-age-distribution/ accessed on 26/01/2023]. In addition, we observed an unbalanced gender distribution in this study, with an unexpected prevalence of the female sex (M/F ratio 0.4). Indeed, the M/F ratio in BD subjects varies from 0.8 to 2.4 in the largest cohorts from the literature, with a similar frequency among men and women in most part of the world or a slight preference for males (7–12). On the other hand, a recent epidemiology study on 1,323 patients from the US reported a 0.3 M/F ratio (13). In this context, given the methodology of this study, we cannot exclude a bias caused by the multiple recruitment channels with a preference for the spontaneous enrolment versus the traditional hospital-based one. However, there is also a chance that our remote recruitment strategy may have allowed a wider inclusion of women in this research compared to the traditional hospital-based recruitment. Indeed, a gender gap in inclusion in clinical studies owing to cultural, biological and economic factors (including necessity to travel) has been widely demonstrated within various patient populations, including rare disease cohorts (14, 15).

The diagnostic delay was around 4 years, similar to what has been observed in historical cohorts of both adults and children with BD (16, 17); however, according to our results, the timeliness of the diagnosis improved over the last decades, reflecting the increasing awareness about BD and general improvement of rare diseases diagnostic paths. Nevertheless, we observed that most patients had to pay for clinical and instrumental exams from their own pocket to achieve the diagnosis of BD, even though the public Italian healthcare system fully covers medical expenses within the rare diseases diagnostic journey. This inconsistency sheds light on the existence of procedural pitfalls of the system, which should be discussed among all the stakeholders to improve the efficacy of existing procedures and introduce new operative measures where required. We also observed that major organ involvement, low socioeconomic status and impaired quality of life are the major determinants of the social burden of BD, in terms of number of accesses to the healthcare system, days of hospitalization and days lost at work by affected people. As for the specific disease manifestations, subjects with gastro-intestinal, neurological, and musculoskeletal manifestations were more likely to access medical services and lose days of work, but also recurrent fever and headache had a remarkable impact on productivity.

According to our data, people affected by BD have medium quality of life (median BDQoL 14, ranging from 0 to 30) and significant level of fatigue (median GFI 38.7, ranging from 1 to 50), in line with the results of a recent systematic review and meta-analysis by Masoumi et al. (18). Quite predictably, quality of life and fatigue were also associated reciprocally and with sport habits, BMI variability and therapy-related necessity/concern perception. A higher impact of physical activity on quality of life in BD has been reported also by Senusi et al. (19). On the other hand, Bodur et al. identified a correlation between disease activity and psychological well-being, measured as Life Satisfaction Index and Nottingham Health Profile, with specific regard to the presence of fatigue, joint involvement, gastro-intestinal involvement, headache and mucosal ulceration (20). Moreover, mucosal, neurological, musculoskeletal and ocular manifestations have been found capable to impact independently on specific SF-36 subscales in an Italian cohort (21). With this respect, the preliminary results of the AIDA for Patients registry confirmed that people complaining of BD-related articular, gastro-intestinal, cutaneous symptoms, headache and fatigue have lower quality of life. However, the measurement of disease activity cannot be separated from the medical examination, which makes necessary to align the patient-driven data collection with the physician-driven prospective records of the AIDA registry. Finally, we found among factors associated to a lower quality of life and fatigue complaint a poor socioeconomic status, a high frequency of medical services and a high work/school absenteeism rate.

Therapeutic adherence has not been studied thoroughly in BD. In an Egyptian cohort, they observed a moderate level of therapeutic adherence measured through the Compliance Questionnaire of Rheumatology (mean CQR score of 69.2 ± 11.79), without identifying statistically significant relationship with sociodemographic or clinical characteristics or the SF-36; on the contrary, they found that the necessity and concern BMQ scores were, respectively, positive and negative predictors of a higher CQR score (22). Our results add further knowledge to the complex evaluation of therapeutic compliance in BD. We calculated a BMQn-c differential of 0.9 ± 1.1 indicating that, when dealing with medications prescribed for BD, the registry participants prioritized necessity belief over concerns to a limited extent. This would conceivably result in a weaker therapeutic adherence, which is in line with further data on therapeutic adherence for patients with BD in the literature (23, 24). Respondents with a more severe disease course characterized by major organ involvement had higher necessity belief than those with minor disease manifestations. Also, more educated participants had higher sense of necessity of treatment, while lower-income people had higher concerns of possible harm from their therapies.

Aligned with the international research agenda on BD, the AIDA for Patients registry may be a key instrument to overcome several barriers identified in the path towards the application of a treat-to-target strategy in everyday clinical practice, including patients’ perceptions about drugs efficacy and safety, socioeconomic aspects like access to healthcare facilities, lack of resources (time, personnel, and financial) required by treat-to-target strategy and adherence to medication (25). However, like all web-based patient-driven registries, the AIDA for patients registry has both advantages and limitations compared to the traditional data collection methodology. The main advantages are the high number of potential study respondents across geographical and cultural boundaries, access to hidden populations and sensitive/ difficult to discuss topics, speed of the participant recruitment and data collection phases, reduced costs, patients’ full and active participation. On the other hand, concerns may arise about sampling issues such as the degree of fit between an online sample and the target population, the reliability of self-reported data, the possibility of multiple submissions and consequent duplication of records, the disparity of access to different web channels and ethical concern for intentional or unintentional misuse (26). These aspects should be considered when applying the results of patient-driven registry-based studies to the general population.

In the case of this study, participants were engaged with the mediation of physicians working in reference centers for BD and a patient advocacy group specifically devoted to BD via mailing list of the association subscribers and its Facebook page. The respondents were directed to a landing page with detailed information on the study aims and inclusion criteria and instructions about how to complete the surveys, in order to mitigate the aforementioned risks of bias. After accepting the study conditions, participants were addressed to a screening survey directly asking whether a definite diagnosis of BD was made by a physician, or the disease was under evaluation or merely suspected by the respondents themselves. Despite these mitigation strategies, we cannot ascertain that all the respondents enrolled via SIMBA communication channels have BD because participants data were fully anonymized. However, the results of the pilot study are consistent with the literature on BD, regardless of the different methodology used, which allowed a consistent sparing of resources in terms of time and dedicated medical personnel. The preliminary analysis of data entered by BD participants suggests that PROs and PREs may be easily provided by the patient remotely, integrating physician-driven registries with complementary and reliable information, which represents one of the major strengths of the AIDA for patient action. In the future, the AIDA for patients instrument will be integrated in the AIDA Network Behçet’s Syndrome Registry to complement it with PROs and PREs directly collected by patients, making them available for clinical research on a wide international cohort. At that stage, it will be also possible to reach the critical numbers allowing comparisons between different recruitment channels to assess in a more comprehensive way the reliability and consistency of data entered by patients themselves.

The AIDA for patients pilot project represents the starting point of a broader initiative that is expected to involve patients affected by autoinflammatory diseases and ocular immune-mediated diseases, their advocates, and caregivers in the next 5 years. Aimed at the development of four-handed registries for clinical research purpose, the project will facilitate interactions among all the figures involved in the co-production of health in all the Countries where AIDA Network partner centers operate. In the light of the AIDA for Patients pilot project experience, the alliance with patient advocates proves itself crucial for the prioritization of the registry domains, for the questionnaire approval, raising awareness, building trust, and getting people actively involved into research.

## Data availability statement

The raw data supporting the conclusions of this article will be made available by the authors, without undue reservation. Requests to access these datasets should be directed to the corresponding author: LC, Research Center of Systemic Autoinflammatory Diseases and Behçet’s Disease Clinics, Department of Medical Sciences, Surgery and Neurosciences, University of Siena, cantariniluca@hotmail.com.

## Ethics statement

The protocol of this study involving human participants was reviewed and approved by the Ethics Committee of Azienda Ospedaliero Universitaria Senese (protocol number: 14951). Written informed consent to participate in this study was provided by the participants or their legal guardian/next of kin electronically at the start of the survey.

## Author contributions

CG designed the study, performed statistical analysis with support from JS, and wrote the first draft of the manuscript. ABi was involved in the registry development as patient representative and enrolled participants via email and social media campaign. LC conceived and designed the study, revised the draft of the manuscript, and accounts for AIDA Registries Coordinator. ABa was involved as bioengineer in the technical development of the registry platform. DR revised the draft of the manuscript. CG, JS, StG, PR, RG, MP, FCr, SM, GEm, AP, AV, MT, VC, RN, VP, CF, and BF enrolled participants in the project during hospital visits. GL, AMai, MC, DR, MG, FL, SeG, PP, AMar, FCi, MM, EA, EBartoloni, AIa, OV, GS, SiG, AIn, EG, GC, PB, AO, ABr, FCa, PT, AMau, GT, AF, HG, GR, ST, JH-R, PS, KL, AK, GEs, FS, HD, AH-A, DO-B, IA, GH, ME, FÖ, EW-S, NA, AT, AS, ŞE, SO, and EBatu were included in the authorship as investigators from the top contributor centres of the AIDA Behçet’s syndrome registry. The authorship was established based on the number of patients recruited in the AIDA registries up to Mar 9th, 2023. All authors contributed to the article and approved the submitted version.

## Funding

This study received funding from the patient advocacy organization S.I.M.B.A (Associazione Italiana Sindrome e Malattia di Behçet).

## Conflict of interest

The authors declare that the research was conducted in the absence of any commercial or financial relationships that could be construed as a potential conflict of interest.

## Publisher’s note

All claims expressed in this article are solely those of the authors and do not necessarily represent those of their affiliated organizations, or those of the publisher, the editors and the reviewers. Any product that may be evaluated in this article, or claim that may be made by its manufacturer, is not guaranteed or endorsed by the publisher.

## Footnotes

^1^https://aidanetwork.org/en/
